# MAP4K4 is a novel MAPK/ERK pathway regulator required for lung adenocarcinoma maintenance

**DOI:** 10.1002/1878-0261.12055

**Published:** 2017-05-02

**Authors:** Xuan Gao, Guangming Chen, Chenxi Gao, Dennis Han Zhang, Shih‐Fan Kuan, Laura P. Stabile, Guoxiang Liu, Jing Hu

**Affiliations:** ^1^ Department of Respiratory Medicine Southwest Hospital Third Military Medical University Chongqing China; ^2^ Department of Pharmacology and Chemical Biology University of Pittsburgh School of Medicine PA USA; ^3^ University of Pittsburgh Cancer Institute PA USA; ^4^ University of Pittsburgh Dietrich School of Arts and Sciences PA USA; ^5^ Department of Pathology University of Pittsburgh School of Medicine PA USA

**Keywords:** cell signaling, EGFR, ERK, lung adenocarcinoma, MAP4K4

## Abstract

About 76% of patients with lung adenocarcinoma harbor activating mutations in the receptor tyrosine kinase (RTK)/RAS/RAF pathways, leading to aberrant activation of the mitogen‐activated protein kinase (MAPK) pathways particularly the MAPK/ERK pathway. However, many lung adenocarcinomas lacking these genomic mutations also display significant MAPK pathway activation, suggesting that additional MAPK pathway alterations remain undetected. This study has identified serine/threonine kinase mitogen‐activated protein 4 kinase 4 (MAP4K4) as a novel positive regulator of MAPK/ERK signaling in lung adenocarcinoma. The results showed that MAP4K4 was drastically elevated in lung adenocarcinoma independently of *KRAS* or *EGFR* mutation status. Knockdown of MAP4K4 inhibited proliferation, anchorage‐independent growth and migration of lung adenocarcinoma cells, and also inhibited human lung adenocarcinoma xenograft growth and metastasis. Mechanistically, we found that MAP4K4 activated ERK through inhibiting protein phosphatase 2 activity. Our results further showed that downregulation of MAP4K4 prevented ERK reactivation in EGFR inhibitor erlotinib‐treated lung adenocarcinoma cells. Together, our findings identify MAP4K4 as a novel MAPK/ERK pathway regulator in lung adenocarcinoma that is required for lung adenocarcinoma maintenance.

**Abbreviations**
EGFR: epidermal growth factor receptor
MAP4K4: mitogen‐activated protein 4 kinase 4
MAPK: mitogen‐activated protein kinase
PP2A: protein phosphatase 2
RTK: receptor tyrosine kinase
shRNA: short hairpin RNA
TMA: tissue microarray

## 1 Introduction

Lung cancer is the most common cause of cancer‐related mortality, and the most common form of lung cancer in the United States among both men and women is adenocarcinoma. The most common somatic aberrations in lung adenocarcinoma are recurrent mutations in the RTK [including epidermal growth factor receptor (EGFR)]/RAS/RAF pathways, which occurred in 76% of cases (Cancer Genome Atlas Research, 2014). RAF serine/threonine kinase phosphorylates MEK to activate MEK, and activated MEK then phosphorylates ERK, leading to its activation (Shaul and Seger, 2007; Wellbrock *et al*., 2004). Because of its pivotal role in cancer genesis and maintenance, the EGFR/RAS/RAF/MEK/ERK signaling cascade has been the subject of intensive research and pharmaceutical scrutiny to identify effective target‐based therapy for cancer treatment (Dhillon *et al*., 2007; Roberts and Der, 2007; Samatar and Poulikakos, 2014). However, the full potential of this signaling pathway as effective targets for cancer therapy has not been realized and many challenges and hurdles remain.

For example, recent clinical trial result showed that the response rate of non‐small‐cell lung cancer patients to vemurafenib (BRAF inhibitor) was 42% (Hyman *et al*., 2015), suggesting that not every BRAF mutant non‐small‐cell lung cancer patients responded to BRAF‐targeted therapy; RAS oncogene is still considered ‘undruggable’, with no effective RAS inhibitors in clinical use; in EGFR‐targeted therapy, although initial response rates are high (71.2–83%) among patients with EGFR mutations, all patients eventually develop resistance (Chong and Janne, 2013). The most common mechanism of resistance to EGFR inhibitors is a secondary mutation in EGFR itself (T790M, threonine‐to‐methionine amino acid change at position 790), which renders the activated kinase insensitive to these agents (Kobayashi *et al*., 2005; Yu *et al*., 2013).

Together, there is an urgent and unmet need for novel therapeutic approaches to effectively block EGFR/RAS/RAF/MEK/ERK signaling. New insights into the regulation of mitogen‐activated protein kinase (MAPK)/ERK signaling pathway could reveal new therapeutic point of intervention, therefore are greatly needed. Given that many lung adenocarcinomas lacking EGFR/RAS/RAF mutations also displayed significant MAPK activation (Cancer Genome Atlas Research, 2014), it is reasonable to predict that there are additional, still undetected MAPK pathway regulators.

The serine/threonine kinase MAP4K4 is a member of the Ste20p (sterile 20 protein) family. MAP4Ks were discovered in 1995 as a key kinase in the mating pathway in *Saccharomyces cerevisiae* (Wu *et al*., 1995). MAP4K4 is best known as an inflammation‐related kinase: MAP4K4 promotes vascular inflammation (Roth Flach *et al*., 2015) and siRNA targeting macrophage MAP4K4 suppresses systemic inflammation (Aouadi *et al*., 2009). Although emerging evidence suggests that MAP4K4 is involved in many aspects of cell functions and processes, information regarding MAP4K4 in different types of cancer is very limited. Results from one previous study showed that MAP4K4 protein level was elevated in lung adenocarcinoma and its elevation was negatively associated with patients’ prognosis (Qiu *et al*., 2012). But except for the correlative observations, whether and how MAP4K4 contributes to lung adenocarcinoma remain to be determined. The current study has identified MAP4K4 as a novel activator of MAPK/ERK signaling in lung adenocarcinoma. The results from functional *in vitro* and *in vivo* studies showed that MAP4K4 was required for the maintenance of the malignant phenotype of lung adenocarcinoma. Our findings suggest that pharmacological inhibition of MAP4K4 could be an effective approach to targeting lung adenocarcinoma, as a stand‐alone therapy or in combination with other treatment.

## 2 Materials and methods

### 2.1 Cell lines and cell culture

All cells were cultured in a 37 °C humidified incubator with 5% CO_2_. A549, H23, H1793, H1650, H1975, and H3255 cell lines were cultured with RPMI‐1640 medium. HEK293T cells were cultured with DMEM. All cell culture medium was supplemented with 5% fetal bovine serum, 100 units·mL^−1^ penicillin, and 100 μg·mL^−1^ streptomycin. BEAS‐2B cells were cultured with bronchial epithelial cell growth medium (Lonza, Allendale, NJ, USA); GA‐1000 was not used.

### 2.2 Plasmid, transient transfection, lentivirus production, and infection

The expression plasmid of HA‐MAP4K4 was constructed by inserting human MAP4K4 sequence into pcDNA3.1‐HA. The expression plasmid of constitutively active ERK2 (plasmid #40819) was purchased from Addgene (Cambridge, MA, USA). Plasmid transfections were performed with Polyjet In Vitro DNA Transfection Reagent (SignaGen Laboratories, Rockville, MD, USA) according to the manufacturer's instruction. Cell lines stably overexpressing MAP4K4 were established by G418 selection. Lentiviral‐based shRNAs targeting MAP4K4 were purchased from Sigma Aldrich (St. Louis, MO, USA). Lentiviruses encoding the shRNA against MAP4K4 or control‐shRNA were produced in HEK293T cells transfected with the lentiviral vector expressing MAP4K4‐shRNA or control‐shRNA using the third‐generation packaging systems (Addgene). The viral particle‐containing media was filtered through syringe filters and subsequently used to infect target cells. Cell lines stably expressing shRNA were established by puromycin selection.

### 2.3 Tissue microarray and immunohistochemistry staining

Human lung adenocarcinoma tissue microarray (TMA) containing 44 cases of lung adenocarcinoma, 44 matched adjacent normal lung tissues, and three cases of normal lung tissues was purchased from US Biomax Inc. (Derwood, MD, USA) TMA slides containing 136 cases of human lung adenocarcinoma with KRAS mutation, EGFR mutation or KRAS, and EGFR wild‐type were prepared as described previously (Villaruz *et al*., 2016). All TMA slides were formalin‐fixed and paraffin‐embedded. Immunohistochemistry (IHC) was performed as described previously (Gao *et al*., 2015). Briefly, paraffin sections were deparaffinized and rehydrated. Antigen retrieval was performed in pressure cooker for 15 min in 20 mm Tris/EDTA buffer (pH 9.0). The sections were incubated overnight with MAP4K4 antibody (Biorbyt, San Francisco, CA, USA) in a humidified chamber at room temperature. After washed with PBS, sections were incubated with HRP‐labeled anti‐rabbit secondary antibody (DAKO Envision+ system, Carpinteria, CA, USA) for 1 h at room temperature. Color visualization was performed with liquid DAB chromogen in imidazole/HCL buffer (pH 7.5) containing hydrogen peroxide until the brown color fully developed. The sections were counterstained with hematoxylin and coverslipped with permanent mounting media. The intensity of TMA staining was scored as 0 (negative), 1 (weak), 2 (moderate), and 3 (strong).

### 2.4 Immunoblotting, antibodies, and reagents

Whole‐cell lysates were prepared in M‐PER buffer supplemented with protease inhibitor and phosphatase inhibitor (Thermo Fisher Scientific, Waltham, MA, USA), resolved by SDS/PAGE, and blotted with indicated antibodies. SuperSignal West Pico Chemiluminescent Substrate and SuperSignal Western Blot Enhancer (Thermo Fisher Scientific) were used to enhance blotting signal when needed. The following antibodies were used in this study: anti‐MAP4K4, anti‐phospho‐ERK1/2, anti‐phospho‐JNK, anti‐JNK, anti‐phospho‐p38, anti‐p38, anti‐AKT, anti‐phospho‐AKT, anti‐MEK1/2, anti‐phospho‐MEK1/2, anti‐RAS and anti‐PP2A‐C (Cell Signaling Technology, Danvers, MA, USA); anti‐ERK1/2, anti‐GAPDH, anti‐β‐actin, anti‐PP2A‐B56β, and anti‐MKP3 (Santa Cruz Biotechnology, Dallas, TX, USA); anti‐6Χ HIS (Immunology Consultants Laboratory, Inc., Lake Oswego, OR, USA). Erlotinib and trametinib were purchased from Selleck Chemicals (Houston, TX, USA). The protein phosphatase 2 (PP2A) inhibitor okadaic acid (OA) was purchased from Sigma Aldrich. The PP2A activator FTY720 was purchased from Cayman Chemical (Ann Arbor, MI, USA). MAP4K4 inhibitor PF‐06260933 was purchased from Tocris Bioscience (Avonmouth, Bristol, UK). EGF was obtained from Thermo Fisher Scientific.

### 2.5 Cell proliferation assay

Cells were seeded in 96‐well plates at a density of 3000 cells per well and incubated at 37 °C for indicated time. Ten microlitre MTT (Sigma Aldrich) was then added into each well. Medium containing MTT was replaced with 100 μL per well DMSO after incubation at 37 °C for 4 h. The absorbance was measured at 490 nm with iMark™ Microplate Absorbance Reader (Bio‐Rad, Hercules, CA, USA). Three independent experiments were performed in triplicate. compusyn software (http://www.combosyn.com), which is based on the Median‐Effect Principle (Chou) and the Combination Index‐Isobologram Theorem (Chou‐Talalay) (Chou, 2010), was employed to calculate the combination index (CI) values, which were used to determine the drug combination effect: CI < 0.75 indicates synergism; CI = 0.75−1.25 indicates additive effects; and CI > 1.25 indicates antagonism.

### 2.6 *In vitro* cell invasion assay

Twelve‐well falcon permeable supports with 8‐μm pores and Matrigel were purchased from Corning Life Sciences (Lowell, MA, USA). The inserts were coated with 300 μg·mL^−1^ Matrigel diluted with cold FBS‐free RPMI‐1640 medium, then placed into 12‐well plates on ice. The Matrigel was allowed to solidify at 37 °C for 3 h. After 1 × 10^5^ indicated cells suspended in 500 μL FBS‐free RPMI‐1640 medium were seeded in the insert chambers, 1 mL RPMI‐1640 with 10% FBS was added into the wells. Cells on the upper surface of the inserts were gently wiped away with PBS‐saturated cotton swabs after incubation at 37 °C for 16–20 h. Cells on the lower surface of the insert were fixed and stained with crystal violet solution (0.05% crystal violet, 1% formaldehyde, and 1% methanol). The stained cells were photographed with a Leica DFC420 C camera connected to a Leica DMI300 B microscope. Three independent experiments were performed in duplicate.

### 2.7 Wound healing assay

Twenty‐four‐well wound healing inserts were purchased from Cell Biolabs, Inc. (San Diego, CA, USA), and used according to the manufacturer's protocol. Briefly, inserts were placed in the 24‐well plates before seeding 5 × 10^5^ indicated cells in each well. The inserts were carefully removed after incubation at 37 °C for 24 h. The cells were rinsed with culture medium and then incubated with 10 μm of mitomycin C (Cayman Chemical) at 37 °C for 2 h. Cells were photographed 0, 24, and 48 h after mitomycin C treatment with a Leica DFC420 C camera connected to a Leica DMI300 B microscope. Percentage of wound area was measured with imagej (https://imagej.net/Downloadsforfree). Three independent experiments were performed in triplicate.

### 2.8 Anchorage‐independent growth assay

The cell lines were trypsinized and 3 × 10^4^ cells were suspended in DMEM containing 0.4% low melting point (LMP) agarose (Promega, Madison, WI, USA). Two milliliter of the above cell suspension was plated over 3 mL bottom agar containing 0.8% LMP agarose in a 60‐mm dish. Two independent experiments were performed in quadruplicate. The dishes were incubated at 37 °C with medium replenished every two days until colony formed. The colonies were stained with 0.01% crystal violet solution. Representative pictures were taken under stereomicroscope (Olympus SZX10, Center Valley, PA, USA).

### 2.9 PP2A phosphatase activity assay

PP2A phosphatase activity was determined using the PP2A immunoprecipitation phosphatase assay kit (Millipore, Billerica, MA, USA) according to the manufacturer's protocol. Briefly, lysates containing 200 μg protein were incubated with 4 μg anti‐PP2A and 40 μL protein A/agarose slurry in Ser/Thr assay buffer for 2 h at 4 °C with constant rocking. After washed with Ser/Thr assay buffer for three times, immunoprecipitated PP2A was incubated with 750 μm phosphopeptide (K‐R‐pT‐I‐R‐R) for 10 min at 30 °C in a shaking incubator. Twenty‐five microlitre of the reaction mixture was transferred into three wells of the 96‐well microtiter plate, and the plate was then incubated with 100 μL of malachite green phosphate detection solution for 15 min at room temperature. Free phosphate was quantified by measuring the absorbance at 650 nm with iMark™ Microplate Absorbance Reader (Bio‐Rad).

### 2.10 RAS activity assay

RAS activity was determined using the RAS activation assay kit (Millipore) according to the manufacturer's protocol. Briefly, the cell lysates were gently rotated with RAF‐1 RBD (RAS binding domain) agarose, which binds to active, GTP‐bound RAS, at 4 °C for 3 h. The precipitates were then subjected to SDS/PAGE and immunoblotted with anti‐RAS antibody.

### 2.11 Animal experiments

All animal procedures were carried out according to the protocols approved by the Institutional Animal Care and Use Committee at the University of Pittsburgh. Four‐ to five‐week‐old nude mice (Charles River) housed in a sterile environment were used for the experiments. For xenograft experiments, 1× 10^6^ H1975 shRNA control cells or MAP4K4‐knockdown cells were subcutaneously injected into the left or right flanks of the mice, respectively. Eight mice were used. Tumor size was determined by caliper measurements twice a week. Tumor volume was calculated using the formula: V = ½ × *a* × *b* ^2^, where *a* and *b* denote the largest and smallest axis of the tumor, respectively. Mice were euthanized 28 days after implantation. Tumors were excised, followed by cryogenic grinding with liquid nitrogen to prepare whole‐cell lysates for immunoblotting with indicated antibodies.

For experimental metastasis, 1 × 10^6^ H1975 shRNA control cells or MAP4K4‐knockdown cells were injected into the tail vein of each mouse, six mice in each group. Mice were euthanized 12 weeks after injection. Mice lungs were fixed with intratracheal instillation of 10% formalin as described previously (Braber *et al*., 2010). Briefly, the caudal vena cava of the animals was cut to prevent the flow of blood into the bases of the lungs. A cannula was inserted into the trachea and fixed with a ligature; 10% formalin was gently injected through the cannula to inflate the lungs. Lungs and heart were removed en bloc and placed in a glass vial filled with 10% formalin. Twenty‐four hours later, 10% formalin was replaced by 70% ethanol. Representative pictures of the lungs were taken. All lungs were embedded by paraffin, and hematoxylin and eosin (H&E) staining was performed. The number of surface lung tumors was counted lobe by lobe while excising the lungs. H&E‐stained slides were scanned by Aperio Digital Pathology Slide Scanner (Leica Biosystems Inc., Buffalo Grove, IL, USA), and tumor diameters were measured with ImageScope.

### 2.12 Statistics

Data were presented as mean ± SD. The difference between two groups was evaluated with Student's *t*‐test (two‐tailed). The difference in the distribution of positively stained TMA samples among three score groups was evaluated with chi‐square test. *P* values less than 0.05 were considered statistically significant.

## 3 Results

### 3.1 MAP4K4 elevation in lung adenocarcinoma is independent of *KRAS* or *EGFR* mutation status

**Figure 1:**
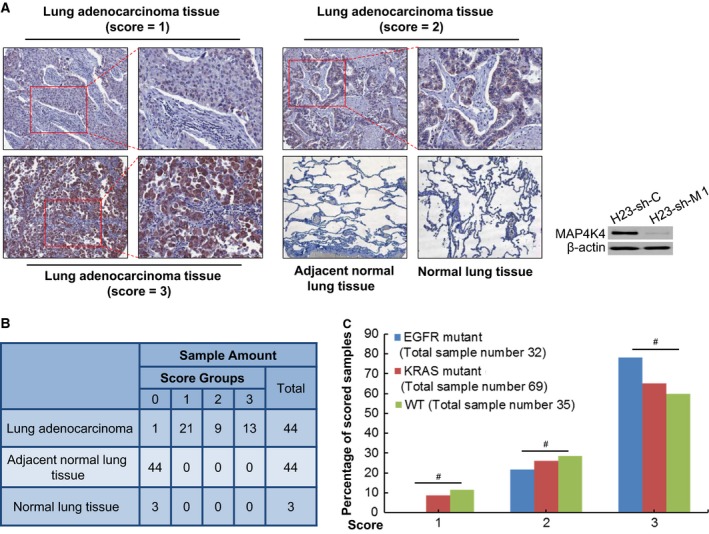
KRAS or EGFR mutation‐independent elevation of MAP4K4 in lung adenocarcinomas. (A,B) Immunohistochemistry (IHC) staining against MAP4K4 was performed on TMA sections containing human lung adenocarcinoma (*n* = 44), adjacent normal lung tissue (*n* = 44), and normal lung tissue (*n* = 3) samples. Staining intensity was scored as 0, negative; 1, weak; 2, moderate; 3, strong. Representative pictures (A, left and middle panels) of distinct levels of MAP4K4 expression and a summary (B) of score distribution of all TMA samples were shown. Immunoblotting was performed to confirm the specificity of anti‐MAP4K4 antibody used in IHC staining (A, right panel). (C) MAP4K4 expression level was measured by IHC staining on TMA sections containing 136 lung adenocarcinoma samples with *EGFR* mutation, *KRAS* mutation, or wild‐type *EGFR* and *KRAS*. TMA slides were scored with the same method used in (A) and (B). Percentage of scored samples in three score groups was shown. *P* value was determined by chi‐square test; ^#^denotes that there was not statistically significant difference (*P* > 0.05).

We performed IHC analysis of MAP4K4 on human lung adenocarcinoma TMAs containing 44 cases of cancer tissues on a slide. Consistent with prior study (Qiu *et al*., 2012), we found that protein expressions of MAP4K4 were nearly undetectable in normal lung tissues and tumor‐adjacent normal tissues but were drastically increased in tumor tissues. Strikingly, nearly 100% of the tumor cells were stained positive for MAP4K4. Representative IHC staining of MAP4K4 is shown in Fig. 1A, and score distributions are summarized in Fig. 1B. Of note, the specificity of anti‐MAP4K4 for IHC was verified by western blot analysis. We then tested whether MAP4K4 elevation was associated with *KRAS* or *EGFR* mutation. The TMA slides, obtained from the University of Pittsburgh Lung Specialized Program of Research Excellence (SPORE) (Villaruz *et al*., 2016) and constructed using randomly selected archival lung adenocarcinomas with known *EGFR* and *KRAS* mutation status, were stained for MAP4K4. As shown in Fig. 1C, the rate of MAP4K4 overexpression did not appear to vary among tumors with *EGFR* mutations (25/32, 78% with MAP4K4 overexpression), *KRAS* mutations (45/69, 65% with MAP4K4 overexpression), or neither mutation (21/35, 60% with MAP4K4 overexpression), implying that MAP4K4 elevation in lung adenocarcinoma is not associated with *KRAS* or *EGFR* mutation, hence excluding mutant KRAS or mutant EGFR as causes for MAP4K4 dysregulation.

### 3.2 MAP4K4 is required for the maintenance of malignant phenotype of lung adenocarcinoma cells

**Figure 2:**
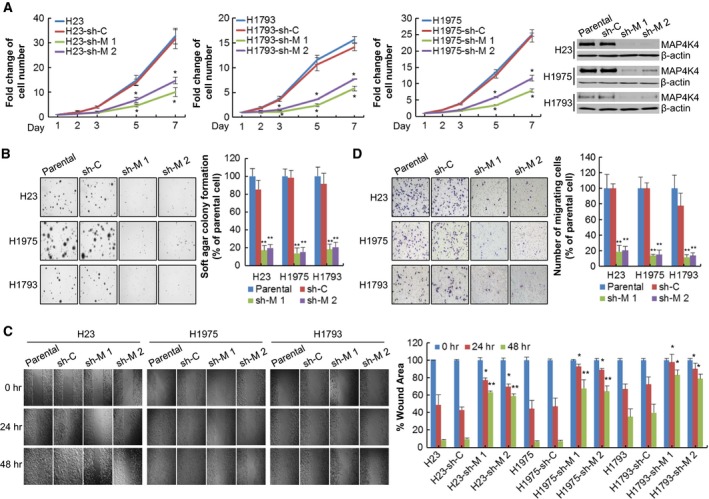
MAP4K4 is important for lung adenocarcinoma cell functions. MAP4K4‐knockdown cell lines or shRNA control cell lines were generated with lentiviral‐based shRNA targeting MAP4K4 (sh‐M 1 and sh‐M 2) or scrambled shRNA (sh‐C) in lung adenocarcinoma cell lines H23 (*KRAS* mutant), H1975 (*EGFR* mutant), and H1793 (*KRAS* and *EGFR* wild‐type). Data in line charts and column charts were shown as means ± SD, * and ** denote a statistically significant difference, *P* < 0.05 and *P* < 0.01, respectively, compared with parental cell lines. (A) Cell viability of different cell lines was measured by MTT assay. MAP4K4 expression was evaluated by immunoblotting (IB) at the time of plating. (B) Left panel: representative pictures of soft agar assay. Right panel: quantification of soft agar assay. (C) Left panel: representative pictures of wound healing assay using 24‐well wound healing inserts. Ten micromolar of mitomycin C was added to each well for 2 h after removing the inserts. Pictures were taken 0, 24, and 48 h after mitomycin C application. Right panel: quantification of wound healing assay. (D) Left panel: representative pictures of *in vitro* cell invasion assay. Right panel: quantification of *in vitro* cell invasion assay.

To determine whether MAP4K4 is functionally involved in lung adenocarcinoma cells, we performed a variety of *in vitro* assays to evaluate the impact of shRNA knockdown of MAP4K4 on cell functions including proliferation, anchorage‐independent growth, migration, and invasion. Three cell lines H23 (*KRAS*‐mutated), H1975 (*EGFR*‐mutated), and H1793 (wild‐type *KRAS* and *EGFR*) were used as cellular models. The results of MTT assays showed that downregulation of MAP4K4 significantly inhibited cell proliferation regardless of *KRAS* or *EGFR* mutation status (Fig. 2A). Furthermore, MAP4K4 knockdown suppressed EGF‐induced growth of H1793 cells (Fig. S1). Downregulation of MAP4K4 also substantially inhibited anchorage‐independent growth of lung adenocarcinoma cells (Fig. 2B). Results from wound healing assay and cell invasion assay further showed that MAP4K4 deficiency jeopardized the abilities of lung adenocarcinoma cells to migrate and to invade (Fig. 2C,D). Next, we examined the effect of MAP4K4‐specific small‐molecule inhibitor, PF‐06260933, on lung adenocarcinoma cell functions. As shown in Fig. S2, treatment of H1975 cells with PF‐06260933 significantly suppressed cell proliferation, anchorage‐independent growth, cell migration, and cell invasion. Together, the above results from *in vitro* cell function analyses suggest that MAP4K4 could be a previously unrealized key player in the regulation of cell functions of lung adenocarcinoma.

### 3.3 MAP4K4 activates the MAPK/ERK pathway in lung adenocarcinoma cells

**Figure 3:**
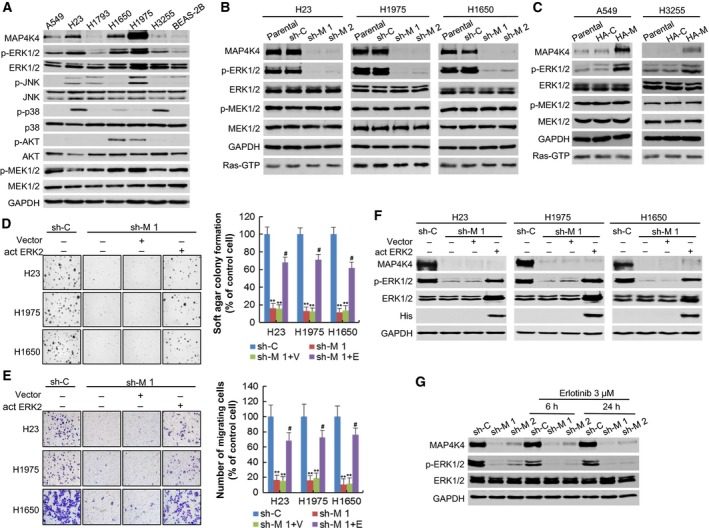
MAPK/ERK1/2 is a downstream signaling mediator of MAP4K4 in lung adenocarcinoma cells. (A) The whole‐cell lysates of different lung adenocarcinoma cell lines, including two *KRAS*‐mutant cell lines, A549 and H23; one *KRAS* and *EGFR* wild‐type cell line, H1793; three *EGFR*‐mutant cell lines, H1650, H1975, and H3255; and one lung bronchus cell line, BEAS‐2B, were used for IB with indicated antibodies. (B) MAP4K4‐knockdown cell lines (sh‐M 1 and sh‐M 2) or shRNA control cell lines (sh‐C) were generated with two different lentiviral‐based shRNA targeting MAP4K4 or scrambled shRNA in H23, H1975, and H1650 cell lines. The whole‐cell lysates were used for IB with indicated antibodies. To detect GTP‐bound RAS, the cell lysates were incubated with RAF‐1 RBD agarose. The bound proteins were then resolved by SDS/PAGE and blotted with anti‐RAS antibody. (C) MAP4K4‐overexpressing cell lines (HA‐M) and control cell lines (HA‐C) were established by transfecting pcDNA3.1‐HA‐MAP4K4 or pcDNA3.1‐HA into A549 or H3255 cell lines followed by G418 selection. The whole‐cell lysates were prepared for IB or subjected to RAS activation assay. (D–F) Constitutively active ERK2 (act ERK2) or vector was transfected into MAP4K4‐knockdown cell lines (sh‐M 1) with Polyjet In Vitro DNA Transfection Reagent. Data in column charts were shown as means ± SD; ** and ^#^ denote a statistically significant difference (*P* < 0.01) and no statistically significant difference (*P* > 0.05), respectively, compared with shRNA control cell lines (sh‐C). (D) Left panel: representative pictures of soft agar assay. Right panel: quantification of soft agar assay. (E) Left panel: representative pictures of *in vitro* cell invasion assay. Right panel: quantification of *in vitro* cell invasion assay. (F) The whole‐cell lysates of different cell lines were used for IB with indicated antibodies. (G) H1975‐sh‐control (sh‐C) and H1975‐sh‐MAP4K4 (sh‐M 1 and sh‐M 2) cells were treated with 3 μm of erlotinib for 6 and 24 h. IB was performed with indicated antibodies.

Identification of downstream signaling mediators of MAP4K4 is crucial for understanding the mechanisms underlying MAP4K4 regulation of lung adenocarcinoma cells. It has been shown that MAP4K4 can induce ERK, JNK, and p38 activation in TNF‐α‐induced signaling (Bouzakri and Zierath, 2007; Bouzakri *et al*., 2009; Tesz *et al*., 2007). Whether these kinases function as MAP4K4 downstream effector in lung adenocarcinoma remains to be determined. To gain initial clues, we compared cellular MAP4K4 levels to the activation status of MAPK/ERK1/2, MEK1/2, MAKP/JNK1/2, MAPK/p38 as well as the serine/threonine protein kinase AKT in lung adenocarcinoma cell lines, evaluated by immunoblotting using specific antibodies against phosphorylated ERK, MEK, JNK, p38, or AKT. The results showed that high cellular levels of MAP4K4 appeared to coincide with high levels of phosphorylated ERK1/2, but not phosphorylation of MEK1/2, JNK, p38, or AKT (Fig. 3A), suggesting that ERK is likely downstream signaling mediator of MAP4K4.

We next took genetic manipulation strategies—shRNA‐knockdown and overexpression approaches – to further validate ERK as MAP4K4 downstream signaling effectors. As shown in Fig. 3B, in cell lines expressing relatively high MAP4K4, shRNA downregulation of MAP4K4 greatly reduced the level of phosphorylated ERK1/2 without changing phosphorylation of MEK and RAS activity (Fig. 3B), indicating that ERK1/2 is the downstream signaling mediator, and regulation of ERK1/2 by MAP4K4 is independent of RAS/MEK/ERK axis. Consistent with this, overexpression of MAP4K4 in cell lines expressing relatively low MAP4K4 substantially increased the level of phosphorylation ERK1/2 without changing MEK phosphorylation and RAS activity (Fig. 3C). Functionally, overexpression of MAP4K4 in these cell lines could also promote cell proliferation (Fig. S3). To further confirm that ERK is a downstream effector of MAP4K4 signaling in lung adenocarcinoma cells, we transfected MAP4K4‐knockdown cells with plasmid expressing constitutively active ERK2^L73P/S151D^, and the results showed that overexpression of active ERK2 antagonized MAP4K4 downregulation‐mediated inhibition of cell functions (Fig. 3D–F). Together, these results identified MAP4K4 as a novel activator of MAPK/ERK pathway in lung adenocarcinoma cells.

To further test that MAP4K4 bypasses MEK to activate ERK, we tested whether overexpression of MAP4K4 prevents MEK inhibitor‐mediated inhibition of ERK. The results showed that ectopic expression of MAP4K4 in A549 cells partially prevented MEK inhibitor trametinib‐mediated inhibition of ERK phosphorylation (Fig. S4A), supporting that MAP4K4 activation could bypass the effect of MEK inhibitor. Consistent with this, we found that concurrent inhibition of MAP4K4 and MEK with specific inhibitors exerted synergistic inhibitory effect on H23 cell proliferation (Fig. S4B).

The most common mechanism for acquired resistance to EGFR inhibitors such as erlotinib is the mutation of threonine 790 to a methionine (T790M) in the kinase domain of the *EGFR* gene, which results in increased kinase affinity for ATP, thus decreasing the sensitivity to ATP‐competitive inhibitors (Yun *et al*., 2008). The current study showed that in T790M‐mutated EGFR‐TKI‐resistant cell line H1975, downregulation of MAP4K4 inhibited ERK activation in the presence and absence of erlotinib treatment (Fig. 3G), implying that MAP4K4 bypasses EGFR to promote ERK activation; that is, MAP4K4 functioned in parallel with EGFR to regulate ERK activation, which in turn suggests that suppressing ERK reactivation through targeting MAP4K4 in lung adenocarcinoma could be a novel strategy to overcome EGFR‐TKI resistance.

### 3.4 MAP4K4 activates ERK1/2 through inhibiting PP2A activity

**Figure 4:**
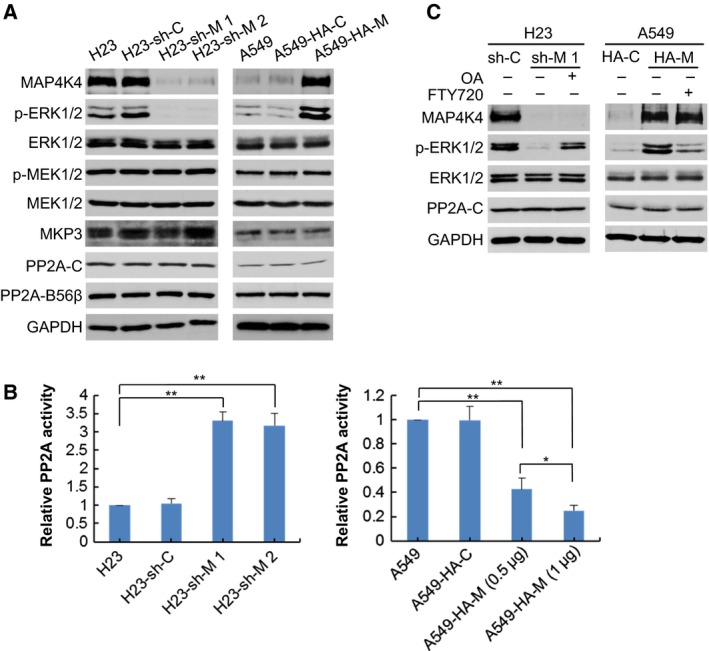
MAP4K4 regulates ERK activation through modulating PP2A activity. (A) The whole‐cell lysates of MAP4K4 shRNA‐knockdown or HA‐overexpressing, shRNA control or HA‐control, and their parental cell lines were used for IB with indicated antibodies. (B) PP2A activity of indicated cells was measured using PP2A immunoprecipitation phosphatase assay kit. * and ** denote a statistically significant difference, *P* < 0.05 and *P* < 0.01, respectively. (C) Whole‐cell lysates of H23 shRNA control cells (sh‐C), H23 MAP4K4‐knockdown cells (sh‐M 1) with and without PP2A inhibitor OA (5 nm) treatment for 24 h beforehand, A549 HA‐control cells (HA‐C) and A549 MAP4K4 HA‐overexpressing cells (HA‐M) with or without PP2A activator FTY720 (2.5 μm) treatment for 24 h beforehand were used for IB with indicated antibodies.

We examined how MAP4K4 regulates ERK activation. As shown in Fig. 4A, shRNA knockdown of MAP4K4 did not inhibit MEK activation (represented as phosphorylated MEK), thus excluding the possibility that MAPK induces ERK activation through MEK. MKP3 (MAP kinase phosphatase 3), also known as dual specificity phosphatase 6 (DUSP6), is the ERK‐specific MAP kinase phosphatase (Muda *et al*., 1996, 1998). Our results show that downregulation of MAP4K4 did not change MKP3 level (Fig. 4A), suggesting that MAP4K4 unlikely regulates ERK activation by modulating MKP3. Phosphatase PP2A is a confirmed tumor suppressor (Sangodkar *et al*., 2016), and it was reported to dephosphorylate both MEK and ERK (Bae and Ceryak, 2009; Letourneux *et al*., 2006; Millward *et al*., 1999; Tay *et al*., 2012). Knockdown of MAP4K4 did not change the protein levels of the PP2A catalytic C subunit and regulatory B56β subunit (Fig. 4A). However, as shown in Fig. 4B, PP2A activity was upregulated in MAP4K4‐knockdown cells and downregulated in MAP4K4‐overexpressing cells. ERK activation can be rescued by PP2A inhibitor OA in MAP4K4‐knockdown cells and suppressed by PP2A activator FTY720 in MAP4K4‐overexpressing cells (Fig. 4C). Together, the above results indicate that MAP4K4 regulates ERK activation through modulating PP2A phosphatase activity.

### 3.5 Downregulation of MAP4K4 inhibits *in vivo* xenograft growth and metastasis of lung adenocarcinoma cells accompanied by suppressed ERK activation

**Figure 5:**
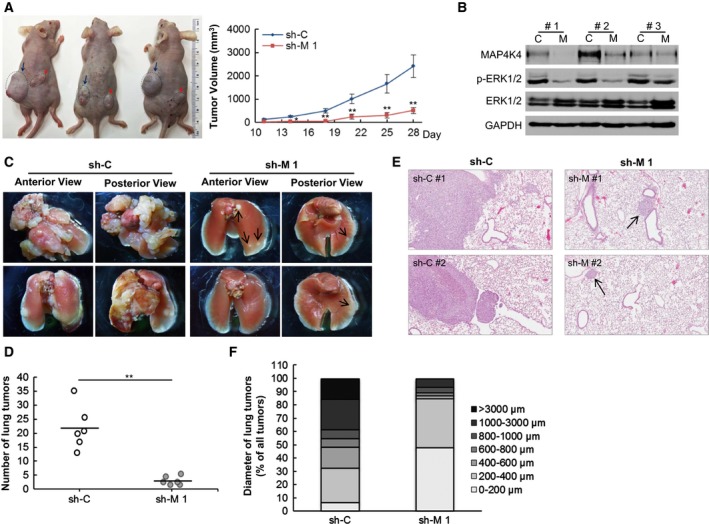
Downregulation of MAP4K4 inhibits *in vivo* xenograft growth and metastasis of lung adenocarcinoma cells. (A,B) 1 × 10^6^ cells of H1975 shRNA control (sh‐C) and MAP4K4 shRNA‐knockdown cell lines (sh‐M 1) were injected subcutaneously into left and right flanks of the same nude mouse (*n* = 8). Mice were sacrificed 28 days after injection. (A) Left panel: picture of representative mice bearing lung adenocarcinoma xenograft. Dark blue arrows and red arrows indicated H1975‐sh‐control and H1975‐sh‐MAP4K4 xenografts, respectively. Right panel: Tumor volume change of each group was presented as means ± SD; * and ** denote a statistically significant difference, *P *< 0.05 and *P* < 0.01, respectively, compared with shRNA control group. (B) IB of three pairs of representative xenograft tumors with indicated antibodies. Each pair was from the same mouse. C: H1975‐sh‐control xenograft; M: H1975‐sh‐MAP4K4 xenograft. (C–F) 1 × 10^6^ cells of H1975‐sh‐control and H1975‐sh‐MAP4K4 cells were injected into tail vein of nude mice, six mice per group. Mice were sacrificed 12 weeks after injection. Lungs of each mouse were dissected and fixed with 10% formalin. (C) Pictures of representative lungs from each group were shown; black arrows indicate metastatic tumors. (D) Total number of surface lung tumors of all mice; ** denotes a statistically significant difference (*P* < 0.01) compared with shRNA control group. (E) Representative pictures of H&E‐stained slides of lung metastatic tumors of each group. (F) The largest diameter of each tumor observed on H&E‐stained slides was measured with ImageScope. The results were subdivided into different size groups. Percentage of the tumors within the group belonging to each subdivision was shown.

We further conducted *in vivo* experiments to validate the functional importance of MAP4K4 in lung adenocarcinoma cells. In xenograft experiments, H1975 shRNA control cells or MAP4K4‐knockdown cells were subcutaneously injected into the left or right flanks of the mice, respectively. As shown in Fig. 5A, knockdown of MAP4K4 in H1975 cells caused significant reduction in tumor size. This result indicated that MAP4K4 is required for *in vivo* xenograft growth of H1975 cells. Immunoblotting analysis of H1975 xenograft tumors revealed that ERK activation was inhibited in MAP4K4‐knockdown cells (Fig. 5B). In experimental metastasis study, H1975 shRNA control cells or MAP4K4‐knockdown cells were injected into the tail vein of each mouse. Results of this assay indicated that knockdown of MAP4K4 greatly impaired the ability of H1975 cells to metastasize to lung (Fig. 5C–F). Together, these findings provided first evidence that MAP4K4 regulates lung adenocarcinoma cell functions *in vivo*, presumably through inducing ERK activation.

## 4 Discussion

To dramatically change the outlook for patients with lung adenocarcinoma, it is essential to identify target that may be exploitable in relapsed *EGFR*‐mutant tumors as well as in lung adenocarcinomas lacking mutations in druggable targets. To this end, the current study has identified MAP4K4 as a previously unrealized ERK pathway activator and it is required for the maintenance of malignant phenotype of lung adenocarcinoma *in vitro* and *in vivo*. Our results indicate that MAP4K4 elevation in lung adenocarcinoma is independent of somatic mutations of *KRAS* or *EGFR*. Importantly, we find that MAP4K4 regulation of MAPK/ERK pathway is also independent of *KRAS* or *EGFR* mutation status, suggesting that MAP4K4‐targeted therapy could be an effective treatment for a broad range of patients with lung adenocarcinoma.

Therapies targeted toward the genetic features of a tumor can be highly effective; therefore, cancer‐relevant mutations/cancer driver genes are considered the best candidates for the development of targeting drugs. However, recent findings suggest that somatically mutated genes are not necessarily the most critical genes for tumor maintenance (Bossi *et al*., 2016). Consistent with this, we have identified MAP4K4 as an import protein required for lung adenocarcinoma maintenance. This finding might have important clinical implications: It suggests MAP4K4‐targeted therapy as a novel treatment option for the *RAS*‐mutated lung adenocarcinoma patients, which currently has few treatment options (Ahearn *et al*., 2012).

MAP4K4 activates MAPK/JNK through TAK1 (a MAP3K) and MKK4/MKK7 (MAP2Ks) in HEK293T cells (Yao *et al*., 1999). In contrast, the current study indicates that MAP4K4 bypasses the three‐tiered kinase canonical cascade (RAF/MEK/ERK) to activate MAPK/ERK by regulating PP2A activity. Regardless of the detailed underlying mechanism, it appears that downstream signaling selection by MAP4K4 and how MAP4K4 activates its target are cell type‐, cancer type‐, or context‐specific.

Several MAP4K4‐specific small‐molecule inhibitors have been available now (Gao *et al*., 2016). However, the potent antitumor properties of these inhibitors remain to be determined. Our novel findings that PF‐06260933 significantly suppressed lung cancer cell functions and had synergistic inhibitory effect with MEK inhibitor on lung cancer cell proliferation suggest that MAP4K4‐specific inhibition could be a promising approach to treating lung adenocarcinoma.

## 5 Conclusions

The current study has identified MAP4K4 as a novel MAPK/ERK activator. Targeting MAP4K4 could be a promising alternative mean of ERK pathway inhibition and MAP4K4 coinhibition could prevent or circumvent resistance to vertical inhibition of EGFR/RAS/RAF/MEK pathway in a broad range of patients with lung adenocarcinoma.

## Data Accessibility

All data generated in this study are provided in the Results section of this article.

## Author contributions

JH and GL contributed to the concept and overall experimental design. SG performed the IHC staining. XG, GC, CG, and DHZ performed the experiments. JH, XG, and CG wrote the manuscript. LPS participated in experimental design and interpretation.

## Supporting information


**Fig. S1.** MAP4K4 knockdown suppresses EGF‐induced lung adenocarcinoma cells growth.

**Fig. S2.** MAP4K4 inhibitor suppresses lung adenocarcinoma cell functions.

**Fig. S3.** MAP4K4 overexpression promotes proliferation of lung adenocarcinoma cells.

**Fig. S4.** Concurrent inhibition of MAP4K4 and MEK exerts synergistic effect on lung adenocarcinoma cells.
