# Supplementary material for: MAP4K4 is a novel MAPK/ERK pathway regulator required for lung adenocarcinoma maintenance
Source: Mol Oncol. 2017 May 2;11(6):628–39. doi: 10.1002/1878-0261.12055 (PMC5467491; doi:10.1002/1878-0261.12055)
Supplement: Supplementary file 1 — Fig. S1. MAP4K4 knockdown suppresses EGF‐induced lung adenocarcinoma cells growth. [file MOL2-11-628-s001.pptx]

## Slide 1
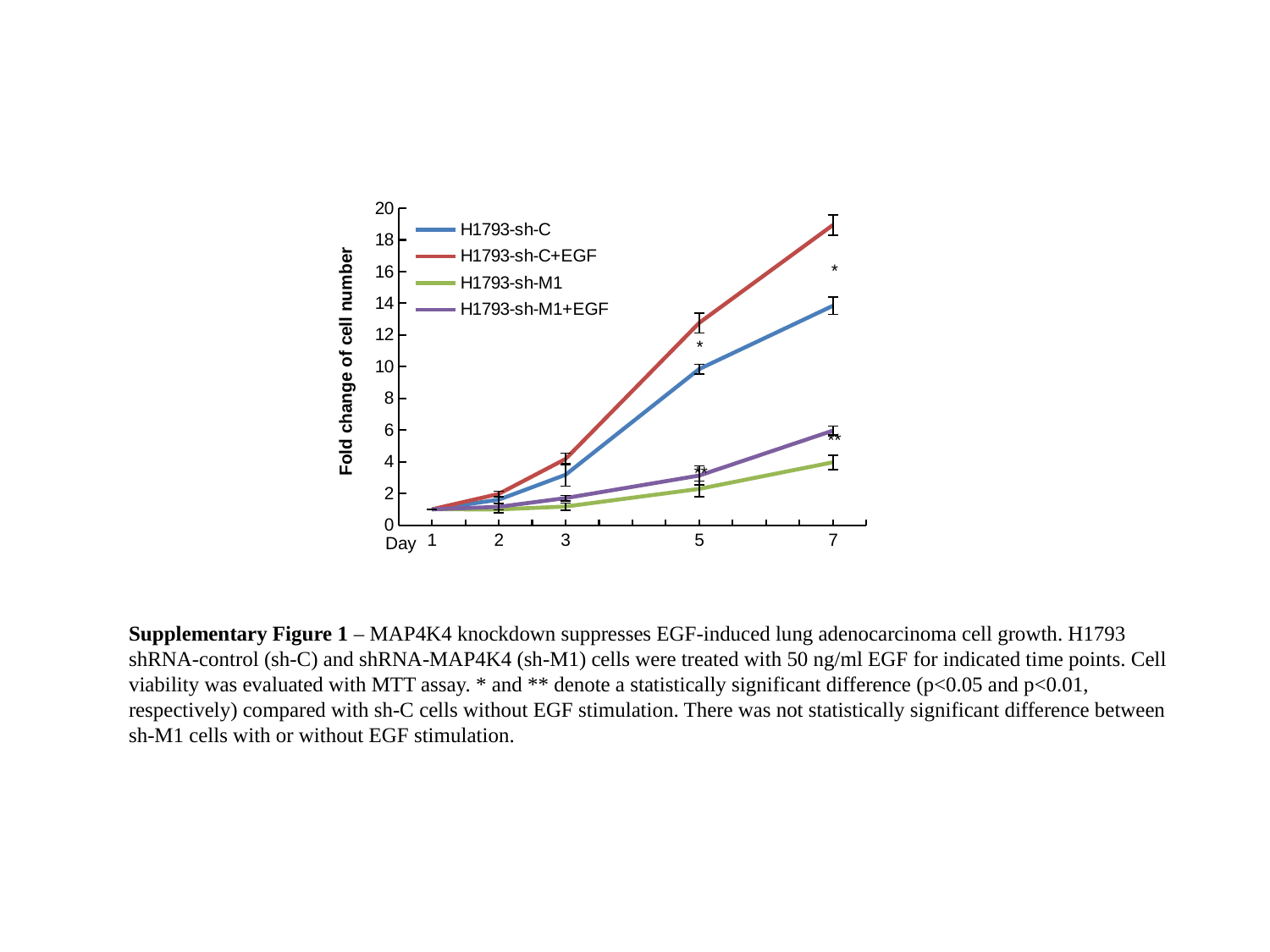

### Chart
| Category | H1793-sh-C | H1793-sh-C+EGF | H1793-sh-M1 | H1793-sh-M1+EGF |
|---|---|---|---|---|
| 1 | 1.0 | 1.0 | 1.0 | 1.0 |
| 2 | 1.6086306952020666 | 1.9736843143416867 | 0.9988828522019038 | 1.16 |
| 3 | 3.1826681907333665 | 4.179260450738384 | 1.1780862026446899 | 1.709666666666667 |
| | None | None | None | None |
| 5 | 9.851904043693432 | 12.766034557096065 | 2.2907115079248865 | 3.131001893483333 |
| | None | None | None | None |
| 7 | 13.845139434388466 | 18.94057715648363 | 3.9662284669847168 | 5.9655 |*
*
**
**
Day
Fold change of cell number
Supplementary Figure 1 – MAP4K4 knockdown suppresses EGF-induced lung adenocarcinoma cell growth. H1793 shRNA-control (sh-C) and shRNA-MAP4K4 (sh-M1) cells were treated with 50 ng/ml EGF for indicated time points. Cell viability was evaluated with MTT assay. * and ** denote a statistically significant difference (p<0.05 and p<0.01, respectively) compared with sh-C cells without EGF stimulation. There was not statistically significant difference between sh-M1 cells with or without EGF stimulation.
