# Supplementary material for: MAP4K4 is a novel MAPK/ERK pathway regulator required for lung adenocarcinoma maintenance
Source: Mol Oncol. 2017 May 2;11(6):628–39. doi: 10.1002/1878-0261.12055 (PMC5467491; doi:10.1002/1878-0261.12055)
Supplement: Supplementary file 2 — Fig. S2. MAP4K4 inhibitor suppresses lung adenocarcinoma cell functions. [file MOL2-11-628-s002.pptx]

## Slide 1
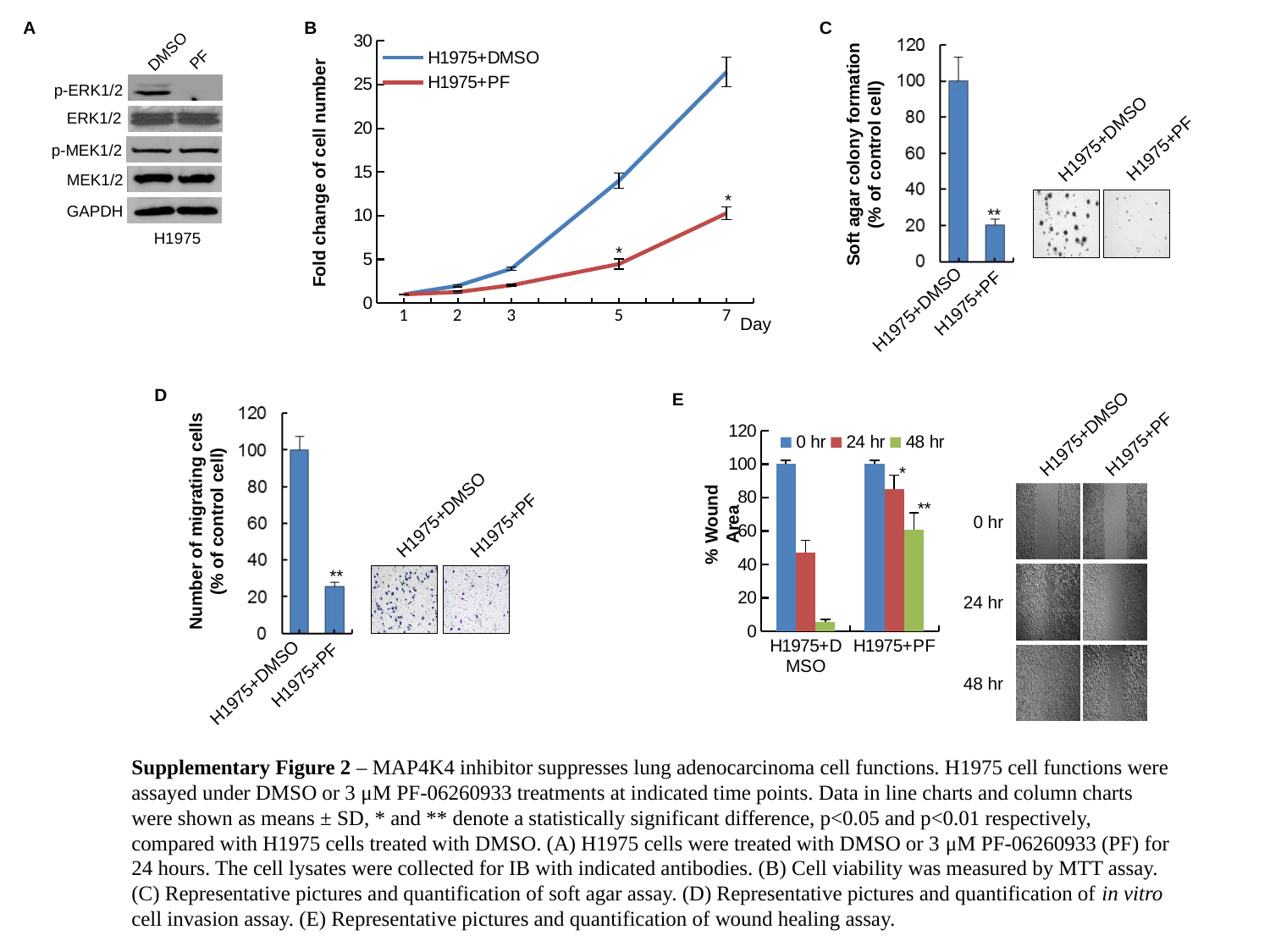

C
Soft agar colony formation
(% of control cell)
**
H1975+PF
H1975+DMSO
H1975+DMSO
H1975+PF
A
B
### Chart
| Category | H1975+DMSO | H1975+PF |
|---|---|---|
| 1 | 1.0 | 1.0 |
| 2 | 1.9738945486973936 | 1.2598931854730433 |
| 3 | 3.9357781681656996 | 2.03754392828976 |
| | None | None |
| 5 | 14.008525969873466 | 4.464692717882527 |
| | None | None |
| 7 | 26.431852636197032 | 10.277768607797102 |Fold change of cell number
*
*
Day
PF
DMSO
p-ERK1/2
ERK1/2
p-MEK1/2
MEK1/2
GAPDH
H1975
Number of migrating cells
(% of control cell)
**
H1975+PF
H1975+DMSO
D
H1975+DMSO
H1975+PF
E
H1975+DMSO
H1975+PF
0 hr
24 hr
48 hr
### Chart
| Category | 0 hr | 24 hr | 48 hr |
|---|---|---|---|
| H1975+DMSO | 100.00000000363909 | 47.30071508023439 | 5.777033789034627 |
| H1975+PF | 100.00000000370899 | 84.87790947051924 | 60.91007379252714 |% Wound Area
*
**
Supplementary Figure 2 – MAP4K4 inhibitor suppresses lung adenocarcinoma cell functions. H1975 cell functions were assayed under DMSO or 3 μM PF-06260933 treatments at indicated time points. Data in line charts and column charts were shown as means ± SD, * and ** denote a statistically significant difference, p<0.05 and p<0.01 respectively, compared with H1975 cells treated with DMSO. (A) H1975 cells were treated with DMSO or 3 μM PF-06260933 (PF) for 24 hours. The cell lysates were collected for IB with indicated antibodies. (B) Cell viability was measured by MTT assay. (C) Representative pictures and quantification of soft agar assay. (D) Representative pictures and quantification of in vitro cell invasion assay. (E) Representative pictures and quantification of wound healing assay.
