# Supplementary material for: MAP4K4 is a novel MAPK/ERK pathway regulator required for lung adenocarcinoma maintenance
Source: Mol Oncol. 2017 May 2;11(6):628–39. doi: 10.1002/1878-0261.12055 (PMC5467491; doi:10.1002/1878-0261.12055)
Supplement: Supplementary file 3 — Fig. S3. MAP4K4 overexpression promotes proliferation of lung adenocarcinoma cells. [file MOL2-11-628-s003.pptx]

## Slide 1
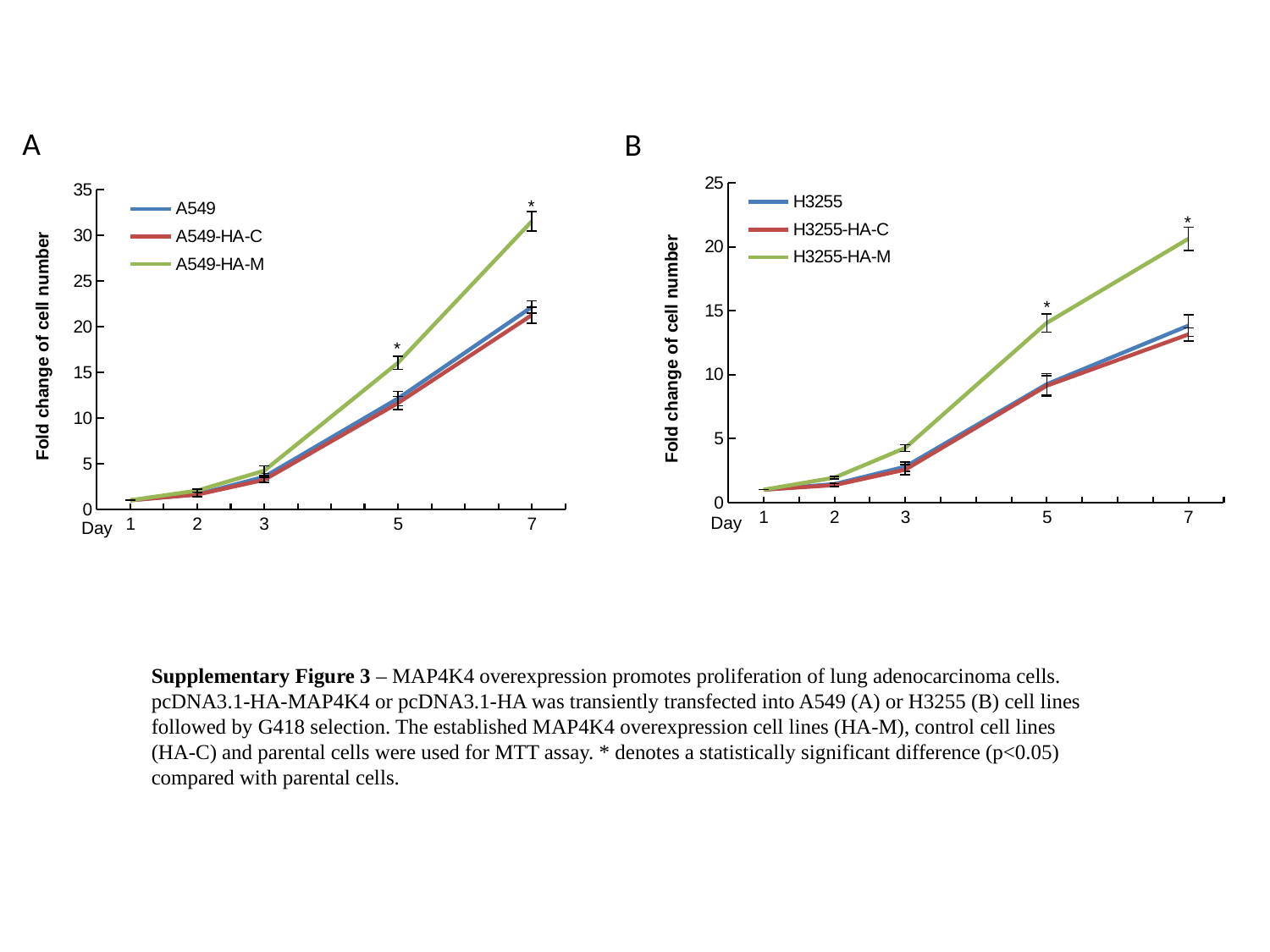

A
B
### Chart
| Category | H3255 | H3255-HA-C | H3255-HA-M |
|---|---|---|---|
| 1 | 1.0 | 1.0 | 1.0 |
| 2 | 1.4347470660107469 | 1.3555210418759334 | 1.9409673590515564 |
| 3 | 2.7951718355312436 | 2.56658211546179 | 4.266695907289367 |
| | None | None | None |
| 5 | 9.25053445838841 | 9.135384512802581 | 14.051839743171966 |
| | None | None | None |
| 7 | 13.836093715300732 | 13.148343769043066 | 20.632895133651335 |*
*
Day
### Chart
| Category | A549 | A549-HA-C | A549-HA-M |
|---|---|---|---|
| 1 | 1.0 | 1.0 | 1.0 |
| 2 | 1.6509092271166665 | 1.6190497761890768 | 2.05343931957197 |
| 3 | 3.5576715041859797 | 3.2330560110441198 | 4.233362573956033 |
| | None | None | None |
| 5 | 12.136008944803232 | 11.638859597665766 | 16.051839743171968 |
| | None | None | None |
| 7 | 22.169427048634066 | 21.2816771023764 | 31.55469863297837 |*
*
Day
Fold change of cell number
Fold change of cell number
Supplementary Figure 3 – MAP4K4 overexpression promotes proliferation of lung adenocarcinoma cells. pcDNA3.1-HA-MAP4K4 or pcDNA3.1-HA was transiently transfected into A549 (A) or H3255 (B) cell lines followed by G418 selection. The established MAP4K4 overexpression cell lines (HA-M), control cell lines (HA-C) and parental cells were used for MTT assay. * denotes a statistically significant difference (p<0.05) compared with parental cells.
