# Supplementary material for: MAP4K4 is a novel MAPK/ERK pathway regulator required for lung adenocarcinoma maintenance
Source: Mol Oncol. 2017 May 2;11(6):628–39. doi: 10.1002/1878-0261.12055 (PMC5467491; doi:10.1002/1878-0261.12055)
Supplement: Supplementary file 4 — Fig. S4. Concurrent inhibition of MAP4K4 and MEK exerts synergistic effect on lung adenocarcinoma cells. [file MOL2-11-628-s004.pptx]

## Slide 1
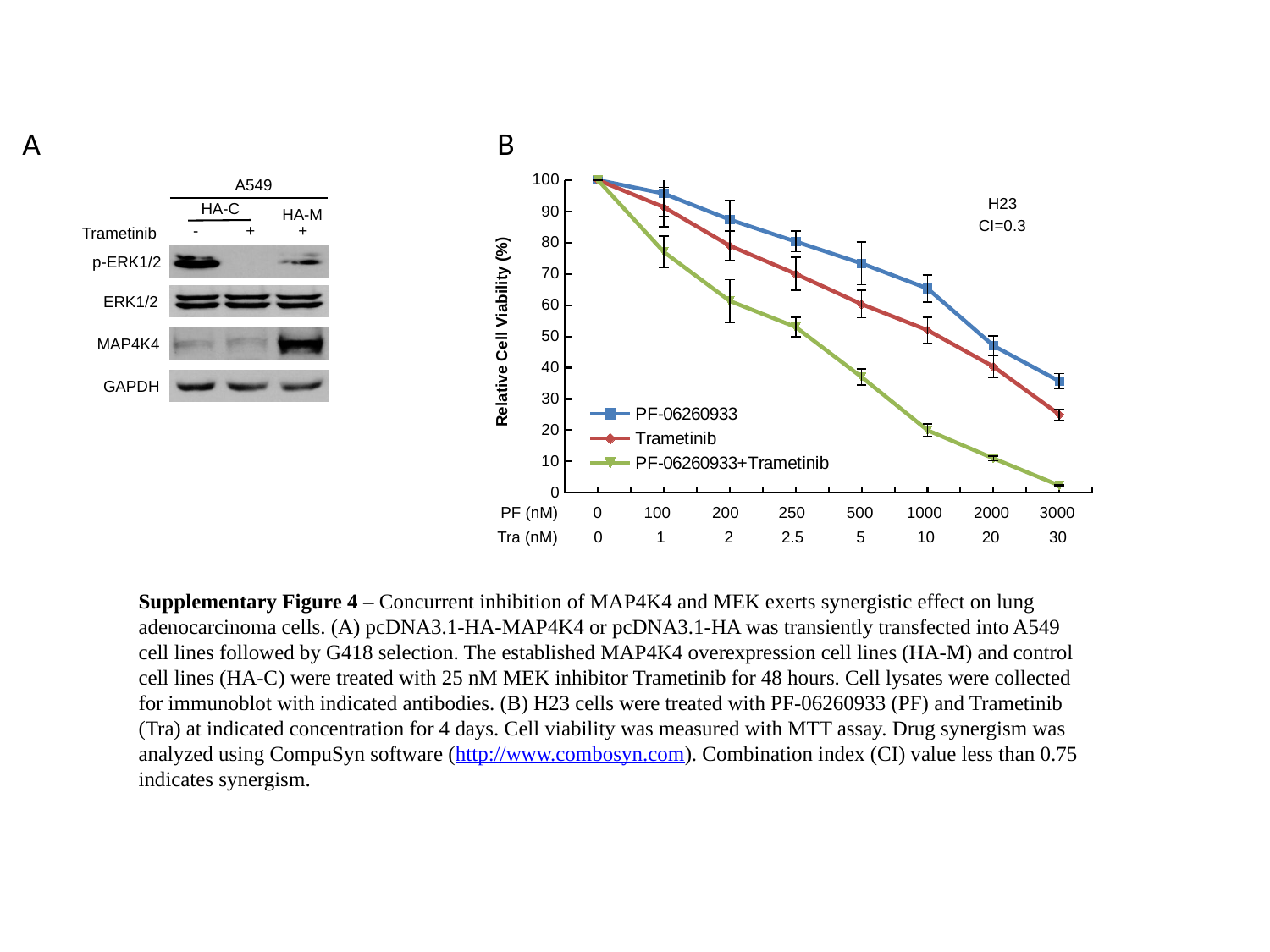

A
B
### Chart
| Category | PF-06260933 | Trametinib | PF-06260933+Trametinib |
|---|---|---|---|
| 0/0 | 100.0 | 100.0 | 100.0 |
| 100/1 | 95.66666666666667 | 91.33333333333333 | 77.0 |
| 200/2 | 87.3333333333333 | 79.0 | 61.33333333333333 |
| 250/2.5 | 80.3333333333333 | 70.0 | 53.0 |
| 500/5 | 73.3333333333333 | 60.333333333333336 | 37.0 |
| 1000/10 | 65.3333333333333 | 52.0 | 20.0 |
| 2000/20 | 47.0 | 40.33333333333333 | 11.0 |
| 3000/30 | 35.666666666666664 | 25.0 | 2.3333333333333335 |H23
Relative Cell Viability (%)
CI=0.3
PF (nM)
0
100
200
250
500
1000
2000
3000
Tra (nM)
0
1
2
2.5
5
10
20
30
A549
HA-C
HA-M
-
+
+
Trametinib
p-ERK1/2
ERK1/2
MAP4K4
GAPDH
Supplementary Figure 4 – Concurrent inhibition of MAP4K4 and MEK exerts synergistic effect on lung adenocarcinoma cells. (A) pcDNA3.1-HA-MAP4K4 or pcDNA3.1-HA was transiently transfected into A549 cell lines followed by G418 selection. The established MAP4K4 overexpression cell lines (HA-M) and control cell lines (HA-C) were treated with 25 nM MEK inhibitor Trametinib for 48 hours. Cell lysates were collected for immunoblot with indicated antibodies. (B) H23 cells were treated with PF-06260933 (PF) and Trametinib (Tra) at indicated concentration for 4 days. Cell viability was measured with MTT assay. Drug synergism was analyzed using CompuSyn software (http://www.combosyn.com). Combination index (CI) value less than 0.75 indicates synergism.
